# SnappySonic: An Ultrasound Acquisition Replay Simulator

**DOI:** 10.5334/jors.289

**Published:** 2020-03-30

**Authors:** Stephen Thompson, Thomas Dowrick, Goufang Xiao, João Ramalhinho, Maria Robu, Mian Ahmad, Dan Taylor, Matthew J. Clarkson

**Affiliations:** Wellcome/EPSRC Centre for Interventional and Surgical Sciences, University College London, London, UK

**Keywords:** ultrasound, simulation, education, aruco, medical device, data visualisation, graphical user interface, GUI, serious games

## Abstract

SnappySonic provides an ultrasound acquisition replay simulator designed for public engagement and training. It provides a simple interface to allow users to experience ultrasound acquisition without the need for specialist hardware or acoustically compatible phantoms. The software is implemented in Python, built on top of a set of open source Python modules targeted at surgical innovation. The library has high potential for reuse, most obviously for those who want to simulate ultrasound acquisition, but it could also be used as a user interface for displaying high dimensional images or video data.

## 1 Introduction

The use of ultrasound acquisition simulators for medical training purposes is increasing and has been shown to provide benefit for training purposes [27, 28]. Removing the need for actual ultrasound saves equipment costs, and allows for more tightly controlled training environments. Currently available ultrasound simulators range from combinations of specialized hardware [6, 14, 15] that provide a tactilely realistic experience to phone based apps [1, 13] that provide convenient training examples. Serious Games for ultrasound training have also been developed using simulated ultrasound images [24] and more abstract representations [20]. However, the available platforms are limited in their configuration options, being tailored to training clinicians on specific anatomical examples. Lack of configuration makes it difficult if you wanted to use your own recorded ultrasound data or configure the geometry to your hardware requirements.

SnappySonic was developed to form part of a public engagement exercise targeted at school age children and their parents/carers. The aim was to demonstrate to the general public (families and our local community) some of the challenges in interpreting ultrasound images. We did this via a serious game, where the user was required to identify objects in recorded ultrasound images. The software provides a semi-realistic experience of ultrasound acquisition, whilst avoiding the need for novice users to cope with such issues as maintaining acoustic contact and beam angle. The software reads a video buffer of appropriate pre-recorded images, and looks up and displays an appropriate image based on the position of a tracked object under the user’s control. Tracking can be done either with a webcam and ArUco markers [25] or with one of NDI’s [7] tracking systems. The user can configure the images to be shown and the geometry of the tracking system. The video buffer we used, together with an example configuration file can be downloaded from the source code repository. The software has been tested by approximately 100 users at a public engagement event, during which we evaluated its performance with a questionnaire, see section 3.1.

## 2 Implementation and architecture

**Figure 1:**
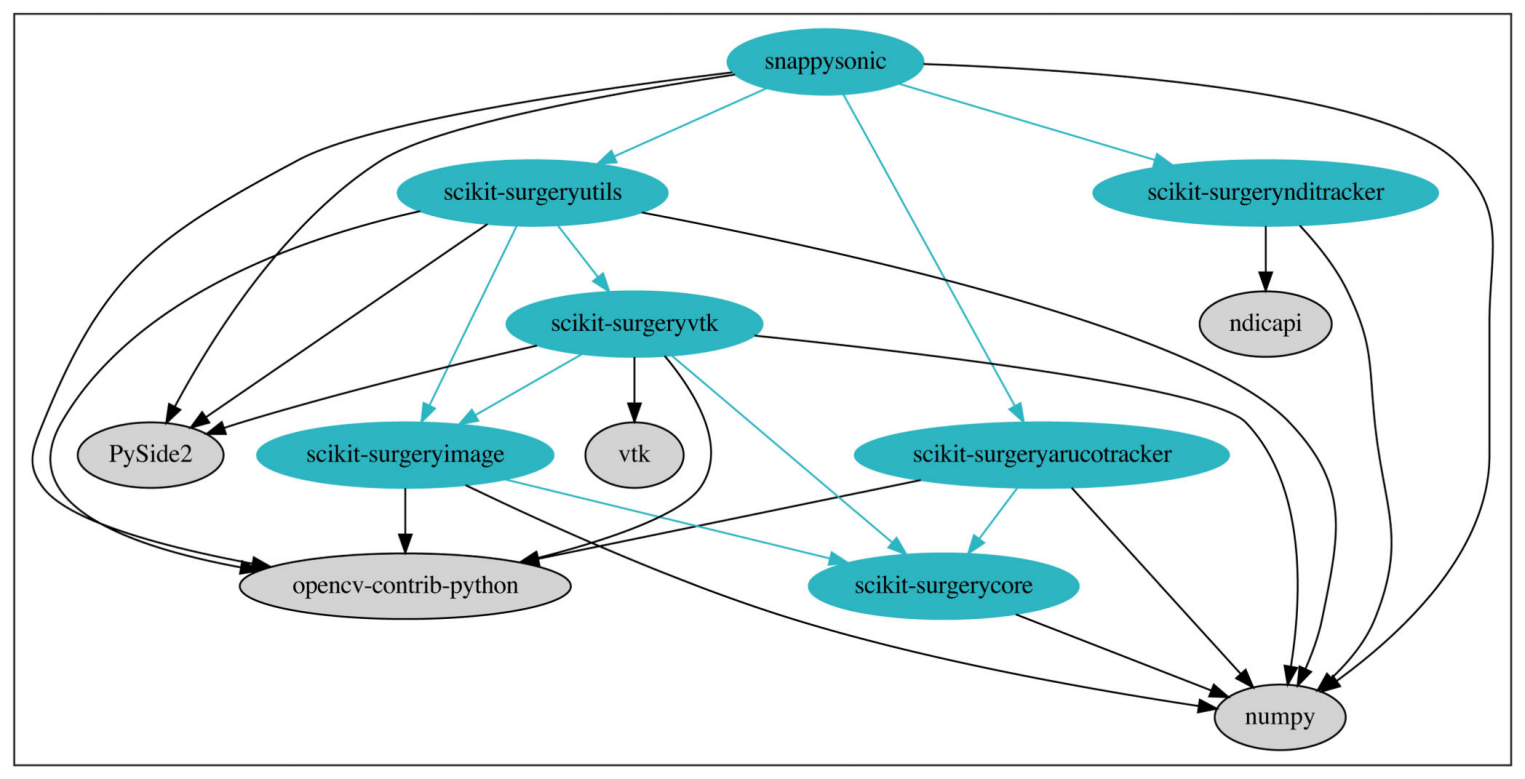
Dependency graph for SnappySonic. All dependencies are from the Python Package index. Dependencies in blue are developed by the authors of this paper and have any further dependencies shown. External dependencies do not have further dependencies shown.

SnappySonic was developed in Python using dependencies available from the Python Packaging Index [12]. Three dependencies are from the SNAPPY [17] software libraries under development by the authors to support innovation in surgical and interventional sciences. There are three further direct dependences on PySide2, NumPy [32] and OpenCV [23]. Figure 1 shows the dependency graph for SnappySonic.

SnappySonic implements an OverlayApp class which inherits from scikit-surgeryutils OverlayBaseApp. OverlayBaseApp implements a Qt widget capable of showing a video image overlaid with a VTK [30] renderer. SnappySonic implements the update member function to take input from a physical tracking system, using scikit-surgerynditracker, or scikit-surgeryarucotracker. The tracker position is used to select an image from an image buffer which is preloaded when the class is initialised. The image buffer used and how the images are selected can be controlled via a configuration file written in JavaScript Object Notation [5]. An image buffer containing ultrasound images of four household items together with an example configuration file can be downloaded from the source repository.

**Figure 2:**
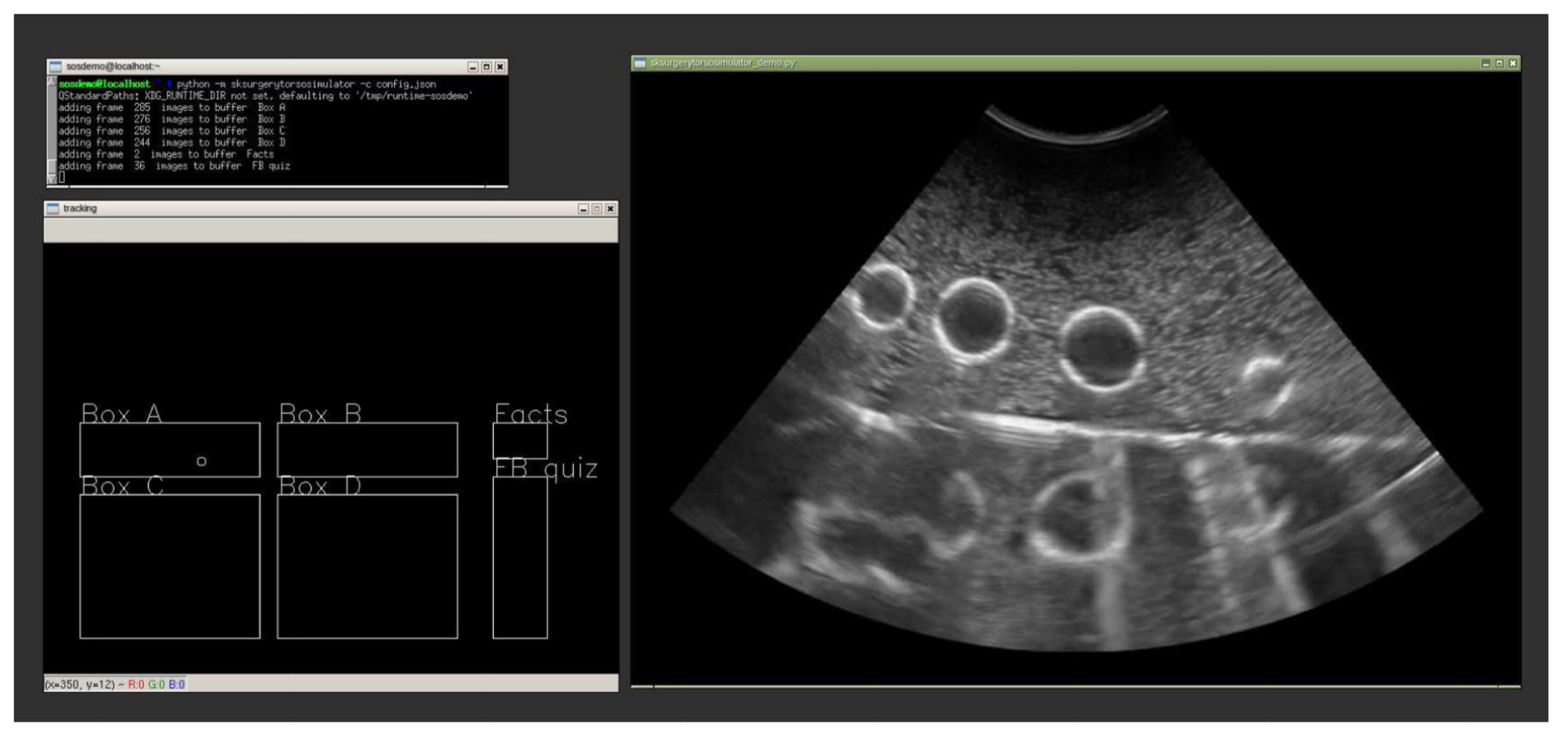
A screen shot of the system in use, top left is the command line and console output. Bottom left a window showing the tracker position with respect to the different parts of the video buffer. At right is the recorded ultrasound image. In this example the image is of a latex glove filled with water, which the user is trying identify by moving the probe around in “Box A”.

Figure 2 shows a screen shot of the software in use. The interface uses two separate windows, one showing the tracking information, so the user knows where they are in the tracked volume, while the second shows the pseudo ultrasound image. It is possible to place the windows on separate screens to prevent the user seeing the tracking information.

**Figure 3:**
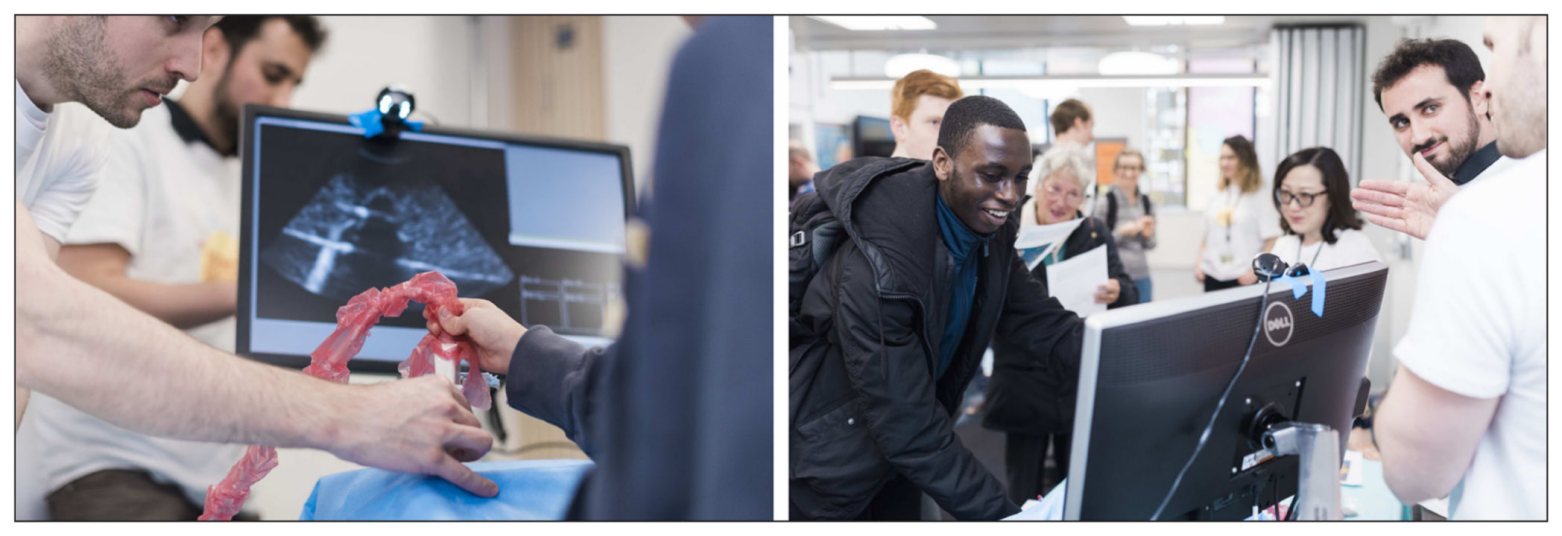
The software in use during our “Science of Surgery” event. We attached a printed ArUco tag to an obsolete ultrasound probe to provide a sense of realism. The user moves the probe over a plastic torso phantom, the probe is tracked by a webcam on top of the monitor, and the ultrasound image shown changes depending on where the probe is over the phantom. Images by James Tye.

Figure 3 shows the system in use, coupled with a plastic torso phantom. We attached an ArUco tag to an obsolete ultrasound probe for a more realistic experience, however it is not necessary to use an ultrasound probe.

## 3 Quality control

SnappySonic, and its dependences (Figure 1) have been developed within the Wellcome/EPSRC Centre for Interventional and Surgical Sciences (WEISS) with the aim to develop robust, reusable libraries to support translational research in surgery, [29]. Well defined software process [31] is central to the development process. WEISS operates its own quality management system (QMS), implementing the IEC EN 62304:2006 standard “Medical device software – Software life cycle processes” [26] to enable the deployment of software to theatre. The software described in this paper falls outside the QMS as it is not a medical device, however we take care to follow as much of the standard as practical, to allow the component software to be used in a medical device at some future date.

We use our own GitLab [3] server [21] for project management, and implement GitLab-CI [4] for continuous integration testing. We use the issue tracker functionality of GitLab to document bug reports and feature requests and reference the issues from code commits. This creates a link between software requirements and development steps, in line with Section 6 of [26].

We use a test driven development process [22]. New features or bug fixes are first defined via a set of failing unit tests. Code changes are then made to get the unit tests passing. GitLab-CI is used to monitor the status of these and existing tests to ensure that any changes do not cause regression of any existing requirements. PyTest [11] is used to manage a suite of unit tests. Tests are executed in different virtual environments, managed using tox [19]. Unit tests are performed independently on individual target environments. Unit test coverage is monitored using coverage [2], where practical a coverage target of 100% is used. There are currently 12 unit tests, providing 100% coverage of the package. 7 of the unit tests cover stand alone functions, while the remaining 5 cover the functioning of the main widget under various configurations. Within the testing framework the Pylint [10] static code analysis tool is used to ensure clear coding style and conformance with PEP 8 [8].

Documentation is generated from the source tree using sphinx [16]. The status of unit tests, coverage, and documentation is communicated to users via flags on the project home page and on the project’s PyPi page.

### 3.1 Functional Testing

**Figure 4:**
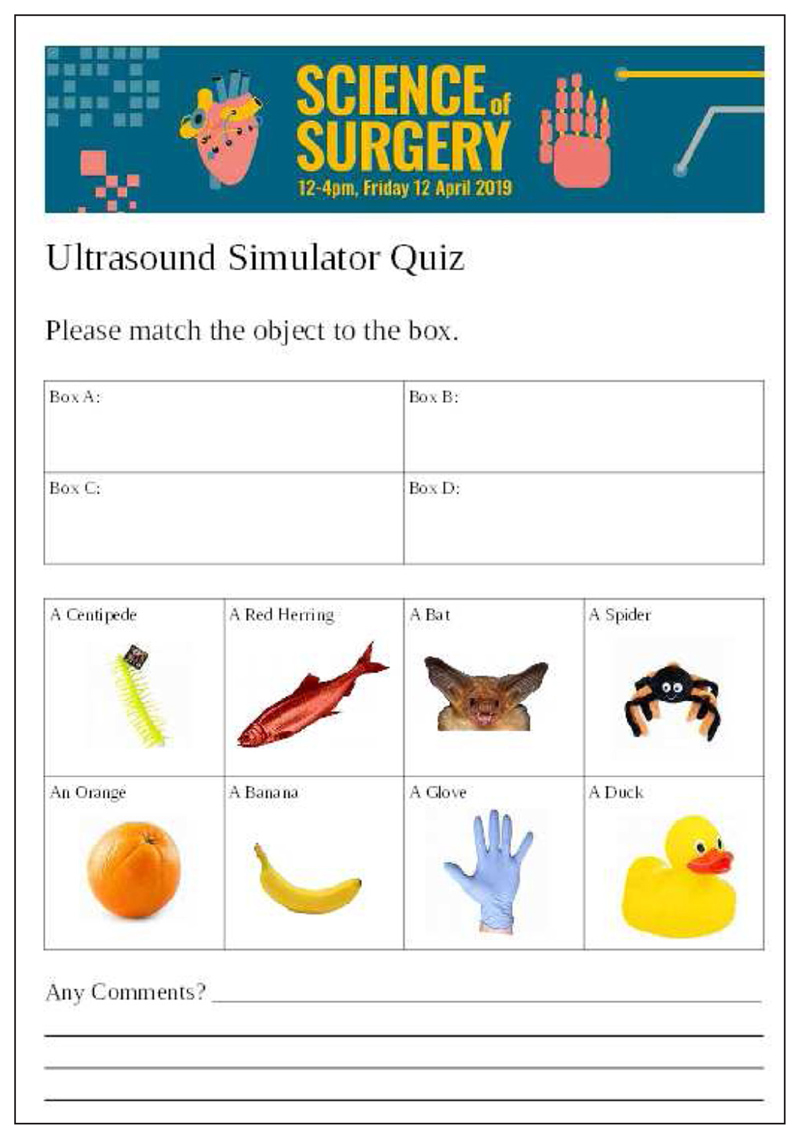
We evaluated the functional performance of the ultrasound simulator during a public engagement event. We asked participants to use the system to identify what household object was “in the box”, from a selection of eight possible objects shown on this form.

The performance of the software was assessed during a public engagement held at WEISS. The ultrasound replay simulator was set up similarly to Figure 3 and members of the public were asked to work out what what objects were “hidden” in boxes, based on interaction with pre-recorded ultrasound images of the objects. A form (Figure 4) was used to determine whether the users had been able to interpret the images correctly. Qualitatively, users agreed that the simulation gave a good experience of ultrasound scanning. Quantitative results are in Table 1.

## 4 Availability and Support

SnappySonic can be installed on supported platforms using the pip [9] installation tool, or downloaded from the source repository [18].

We welcome feature requests and bug reports, which can be submitted via the source repository’s [18] issue tracker, or by emailing the lead author.

### 4.1 Operating system

SnappySonic is available for Python 3.6 onwards and has been tested on Linux, Windows, and MacOS.

### 4.2 Programming language

Python 3.6

### 4.3 Additional system requirements

A webcam is required to use the ArUco based tracking system.

### 4.4 Dependencies

Excluding the scikit-surgery libraries listed in Figure 1, SnappySonic has the following external dependencies numpy>=1.11 opencv-contrib-python>=3.4.4 PySide2<=5. 11.0 vtk.

### 4.5 List of contributors

The contributors are the listed authors.

### 4.6 Software location

#### 4.6.1 Archive

***Name:*** SnappySonic

***Persistent identifier:*** 10.5281/zenodo.3491054

***Licence:*** BSD Licence

***Publisher:*** Zenodo

***Version published:*** v0.0.2

***Date published:*** 05/09/19

#### 4.6.2 Code repository

***Location:*** https://weisslab.cs.ucl.ac.uk/WEISS/SoftwareRepositories/SNAPPY/scikit-surgerytorsosimulator/

***Licence:*** BSD Licence

***Date published:*** 31/07/19

### 4.7 Language

English

## 5 Reuse potential

SnappySonic can most obviously be reused by anyone who wants to create a customisable ultrasound acquisition simulator, either using the ultrasound data buffer within the source repository or by recording their own data. However reuse is not limited to ultrasound data. The interface could be used to navigate quickly through any image buffer so may have applications in video navigation or navigation though multidimensional medical image data sets.

The dependent libraries can also be reused individually or in combination. The software may be forked via gitlab which implements issue trackers to enable bug reporting and feature requests.

## Figures and Tables

**Table 1:** The results of the functional test. 35 users filled in the form. Most users were able to correctly identify objects based on the recorded ultrasound. The orange was notably more challenging to identify.

	Box A: Glove	Box B: Centipede	Box C: Duck	Box D: Orange

No. Right	28	27	28	20
No. Wrong	7	8	7	15
